# Convergence of infectious and non-communicable disease epidemics in rural South Africa: a cross-sectional, population-based multimorbidity study

**DOI:** 10.1016/S2214-109X(21)00176-5

**Published:** 2021-06-15

**Authors:** Emily B Wong, Stephen Olivier, Resign Gunda, Olivier Koole, Ashmika Surujdeen, Dickman Gareta, Day Munatsi, Tshwaraganang H Modise, Jaco Dreyer, Siyabonga Nxumalo, Theresa K Smit, Greg Ording-Jespersen, Innocentia B Mpofana, Khadija Khan, Zizile E L Sikhosana, Sashen Moodley, Yen-Ju Shen, Thandeka Khoza, Ngcebo Mhlongo, Sanah Bucibo, Kennedy Nyamande, Kathy J Baisley, Diego Cuadros, Frank Tanser, Alison D Grant, Kobus Herbst, Janet Seeley, Willem A Hanekom, Thumbi Ndung'u, Mark J Siedner, Deenan Pillay, Vukuzazi Team, Emily B. Wong, Stephen Olivier, Resign Gunda, Olivier Koole, Ashmika Surujdeen, Dickman Gareta, Day Munatsi, Tswaraganang H. Modise, Jaco Dreyer, Siyabonga Nxumalo, Theresa K. Smit, Greg Ording-Jespersen, Innocentia B. Mpofana, Khadija Khan, Zizile E.L. Sikhosana, Sashen Moodley, Yen-Ju Shen, Thandeka Khoza, Ngcebo Mhlongo, Sana Bucibo, Kennedy Nyamande, Kathy J. Baisley, Diego Cuadros, Frank Tanser, Alison D. Grant, Kobus Herbst, Janet Seeley, Willem A. Hanekom, Thumbi Ndung'u, Mark J. Siedner, Deenan Pillay, Mosa Suleman, Jaikrishna Kalideen, Ramesh Jackpersad, Kgaugelo Moropane, Boitsholo Mfolo, Khabonina Malomane, Hlolisile Khumalo, Nompilo Buthelezi, Nozipho Mbonambi, Hloniphile Ngubane, Thokozani Simelane, Khanyisani Buthelezi, Sphiwe Ntuli, Nombuyiselo Zondi, Siboniso Nene, Bongumenzi Ndlovu, Talente Ntimbane, Mbali Mbuyisa, Xolani Mkhize, Melusi Sibiya, Ntombiyenkosi Ntombela, Mandisi Dlamini, Hlobisile Chonco, Hlengiwe Dlamini, Doctar Mlambo, Nonhlahla Mzimela, Zinhle Buthelezi, Zinhle Mthembu, Thokozani Bhengu, Sandile Mtehmbu, Phumelele Mthethwa, Zamashandu Mbatha, Welcome Petros Mthembu, Anele Mkhwanazi, Mandlakayise Sikhali, Phakamani Mkhwanazi, Ntombiyenhlahla Mkhwanazi, Rose Myeni, Fezeka Mfeka, Hlobisile Gumede, Nonceba Mfeka, Ayanda Zungu, Hlobisile Gumede, Nonhlahla Mfekayi, Smangaliso Zulu, Mzamo Buthelezi, Senzeni Mkhwanazi, Mlungisi Dube, Philippa Matthews, Siphephelo Dlamini, Hosea Kambonde, Lindani Mthembu, Seneme Mchunu, Sibahle Gumbi, Tumi Madolo, Thengokwakhe Nkosi, Sibusiso Mkhwanazi, Simbusio Nsibande, Mpumelelo Steto, Sibusiso Mhlongo, Velile Vellem, Pfarelo Tshivase, Jabu Kwinda, Bongani Magwaza, Siyabonga Nsibande, Skhumbuzo Mthombeni, Sphiwe Clement Mthembu, Antony Rapulana, Jade Cousins, Thabile Zondi, Nagavelli Padayachi, Freddy Mabetlela, Simphiwe Ntshangase, Nomfundo Luthuli, Sithembile Ngcobo, Kayleen Brien, Sizwe Ndlela, Nomfundo Ngema, Nokukhanya Ntshakala, Anupa Singh, Rochelle Singh, Logan Pillay, Kandaseelan Chetty, Asthentha Govender, Pamela Ramkalawon, Nondumiso Mabaso, Kimeshree Perumal, Senamile Makhari, Nondumiso Khuluse, Nondumiso Zitha, Hlengiwe Khati, Mbuti Mofokeng, Nomathamsanqa Majozi, Nceba Gqaleni, Hannah Keal, Phumla Ngcobo, Costa Criticos, Raynold Zondo, Dilip Kalyan, Clive Mavimbela, Anand Ramnanan, Sashin Harilall

**Affiliations:** a Africa Health Research Institute, KwaZulu-Natal, Durban, South Africa; b Division of Infectious Diseases, Massachusetts General Hospital, Boston, MA, USA; c Division of Infectious Diseases, University of Alabama Birmingham, Birmingham, AL, USA; d Division of Infection and Immunity, University College London, London, UK; e School of Nursing and Public Health, College of Health Sciences, University of KwaZulu-Natal, Durban, South Africa; f School of Clinical Medicine, College of Health Sciences, University of KwaZulu-Natal, Durban, South Africa; g London School of Hygiene & Tropical Medicine, London, UK; h Department of Pulmonology and Critical Care, Inkosi Albert Luthuli Hospital, Durban, South Africa; i Department of Respiratory Medicine, Nelson R Mandela School of Medicine, University of KwaZulu-Natal, Durban, South Africa; j HIV Pathogenesis Programme, Doris Duke Medical Research Institute, Nelson R Mandela School of Medicine, University of KwaZulu-Natal, Durban, South Africa; k Department of Geography, University of Cincinnati, USA; l Lincoln International Institute for Rural Health, University of Lincoln, UK; m Centre for the AIDS Programme of Research in South Africa, University of KwaZulu-Natal, Durban, South Africa; n School of Public Health, University of the Witwatersrand, Johannesburg, South Africa; o Department of Science and Innovation, Medical Research Council, South African Population Research Infrastructure, Durban, South Africa; p The Ragon Institute of Massachusetts General Hospital, Massachusetts Institute of Technology and Harvard Medical School, Cambridge, MA, USA; q Max Planck Institute for Infection Biology, Berlin, Germany

## Abstract

**Background:**

There has been remarkable progress in the treatment of HIV throughout sub-Saharan Africa, but there are few data on the prevalence and overlap of other significant causes of disease in HIV endemic populations. Our aim was to identify the prevalence and overlap of infectious and non-communicable diseases in such a population in rural South Africa.

**Methods:**

We did a cross-sectional study of eligible adolescents and adults from the Africa Health Research Institute demographic surveillance area in the uMkhanyakude district of KwaZulu-Natal, South Africa. The participants, who were 15 years or older, were invited to participate at a mobile health camp. Medical history for HIV, tuberculosis, hypertension, and diabetes was established through a questionnaire. Blood pressure measurements, chest x-rays, and tests of blood and sputum were taken to estimate the population prevalence and geospatial distribution of HIV, active and lifetime tuberculosis, elevated blood glucose, elevated blood pressure, and combinations of these.

**Findings:**

17 118 adolescents and adults were recruited from May 25, 2018, to Nov 28, 2019, and assessed. Overall, 52·1% (95% CI 51·3–52·9) had at least one active disease. 34·2% (33·5–34·9) had HIV, 1·4% (1·2–1·6) had active tuberculosis, 21·8% (21·2–22·4) had lifetime tuberculosis, 8·5% (8·1–8·9) had elevated blood glucose, and 23·0% (22·4–23·6) had elevated blood pressure. Appropriate treatment and optimal disease control was highest for HIV (78·1%), and lower for elevated blood pressure (42·5%), active tuberculosis (29·6%), and elevated blood glucose (7·1%). Disease prevalence differed notably by sex, across age groups, and geospatially: men had a higher prevalence of active and lifetime tuberculosis, whereas women had a substantially high prevalence of HIV at 30–49 years and an increasing prevalence of multiple and poorly controlled non-communicable diseases when older than 50 years.

**Interpretation:**

We found a convergence of infectious and non-communicable disease epidemics in a rural South African population, with HIV well treated relative to all other diseases, but tuberculosis, elevated blood glucose, and elevated blood pressure poorly diagnosed and treated. A public health response that expands the successes of the HIV testing and treatment programme to provide multidisease care targeted to specific populations is required to optimise health in such settings in sub-Saharan Africa.

**Funding:**

Wellcome Trust, Bill & Melinda Gates Foundation, the South African Department of Science and Innovation, South African Medical Research Council, and South African Population Research Infrastructure Network.

**Translation:**

For the isiZulu translation of the abstract see Supplementary Materials section.

## Introduction

15 years after massive public health efforts to increase access to antiretroviral therapy across sub-Saharan Africa, the rate of HIV-associated mortality is dropping and life expectancy is rising.^1^, ^2^ This shift and ongoing demographic transitions from largely rural to more urban and sedentary lifestyles are increasing risk factors for cardiovascular and metabolic non-communicable diseases across the continent.^3^ Consequently, integrated strategies to prevent and treat HIV that also address other causes of disease, including tuberculosis and non-communicable diseases, are of increasing priority.^4^, ^5^, ^6^ Multimorbidity, defined as multiple medical conditions occurring simultaneously in a single individual, is increasing globally.^7^ Data on the patterns, joint risk factors, and outcomes of multimorbidity across sub-Saharan African populations are few because many cohorts in the region are either hospital-based or clinic-based, or because these data focus on specific diseases or demographic subpopulations.^8^, ^9^, ^10^, ^11^ The successful design of public health programmes requires a clear understanding of the effect and interaction of multiple infectious and chronic conditions.

**Research in context**

**Evidence before this study**

Across sub-Saharan Africa, mortality related to HIV is dropping because of widespread antiretroviral therapy use over the last 15 years. This change is driving increasing life expectancy and decreasing rates of tuberculosis. However, morbidity and mortality from non-communicable diseases, including cardiovascular disease, diabetes, and chronic post-tuberculosis lung disease, are rising in the region. In this context, there is an increasing concern about the effect of multimorbidity or the overlap of two or more chronic conditions on health in African settings. Data from large systematic sources, including the Global Burden of Disease studies, have shown these trends. More granular and localised data are available through health systems data, but these sources do not capture members of the population who have not accessed the health system. Large population-based studies of HIV and non-communicable diseases in Kenya, Uganda, and Karonga, Malawi, have shown the high prevalence of uncontrolled high blood pressure and diabetes in populations with a high HIV prevalence. To date, population-based surveys that include the characterisation of HIV and non-communicable disease in South Africa, the country with the world's largest HIV epidemic, have been limited to either specific conditions or specific age categories, or have not included tuberculosis. To inform the design of the protocol, we searched PubMed for original reports or reviews using the search terms “HIV” OR “HIV infections”, “TB” OR “tuberculosis”, “noncommunicable diseases”, “Africa”, and “multimorbidity” (search done between Nov 1, 2016, and April 27, 2017).

**Added value of this study**

In a population in rural KwaZulu-Natal, South Africa that has been under continuous demographic surveillance for 20 years, we used mobile health camps to measure blood pressure, glycosylated haemoglobin, HIV serology, and HIV viral load, and used a digital chest x-ray and sputum tests for *Mycobacterium tuberculosis,* to simultaneously define HIV, active and lifetime tuberculosis, elevated blood glucose, and elevated blood pressure in more than 17 000 adolescents and adults. On the basis of knowledge of the underlying population structure, we estimated age-specific and sex-specific population prevalence rates of each disease, the extent to which each disease was optimally diagnosed and treated, and the prevalence of multimorbidity using a scale that combined the number of simultaneous diseases and their state of control. Geospatial and lifespan analysis of individual diseases and multimorbidity showed distinct prevalence patterns for HIV and multimorbidity.

**Implications of all the available evidence**

In this population and others in sub-Saharan Africa, although HIV diagnosis and the uptake of antiretroviral therapy has not reached the Joint United Nations Programme on HIV and AIDS's 90–90–90 goals (that by 2020, 90% of individuals positive for HIV will be diagnosed, 90% of individuals diagnosed will be on antiretroviral therapy, and 90% of people on antiretroviral therapy will be virally suppressed), the majority of HIV-positive individuals are on effective chronic treatment. In contrast, most people with active tuberculosis, elevated blood pressure, and elevated blood glucose have undiagnosed or suboptimally treated disease. Women bear a particularly high burden of HIV, elevated blood pressure, and elevated blood glucose. Men have higher rates of active and lifetime tuberculosis, putting them at risk for chronic lung disease. In the absence of a rapid improvement of public health systems to prevent, diagnose, and treat these diseases, the burden of multimorbidity will worsen as the cohort of men and women with the highest rates of HIV infection (currently in their fourth and fifth decades of life) get older over the next several decades. Population-based studies of the convergence of infectious and non-communicable diseases are needed to inform the design of interventions to address multimorbidity in sub-Saharan Africa and other low-income and middle-income settings.

Our aim was to measure the prevalence and overlap of four common and treatable infectious and non-communicable diseases in an HIV endemic population. Additionally, we aimed to establish the degree to which individual diseases and multimorbidity were diagnosed and optimally managed in the population. To do so, we created a new research programme, Vukuzazi (“wake up and know ourselves” in isiZulu), leveraging an existing demographic and health surveillance cohort that has yielded substantial information about HIV transmission and epidemic dynamics over the past 20 years.^12^, ^13^ We did community-based disease phenotyping for HIV, tuberculosis, elevated blood pressure, and elevated blood glucose, and analysed demographic and geospatial patterns of multimorbidity to inform future public health and scientific priorities in HIV endemic African populations.

## Methods

### Study setting and recruitment

**Figure 1:**
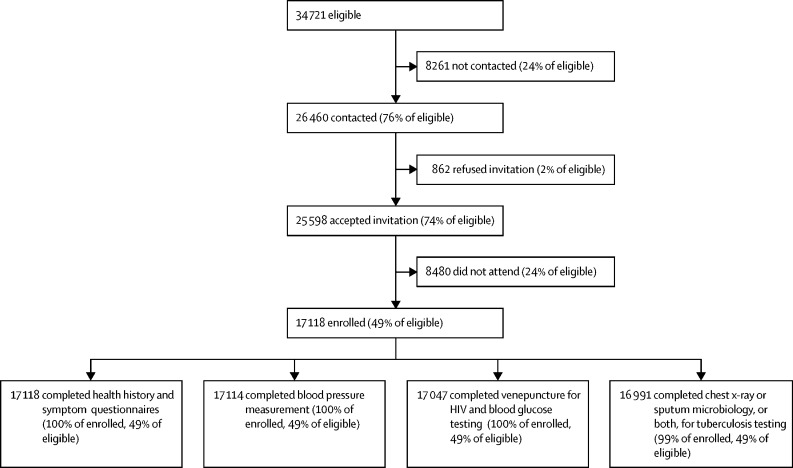
Participation in the Vukuzazi study Individuals aged 15 years or older and living in the Africa Heath Research Institute Demographic and Health Surveillance area were eligible for Vukuzazi. The flow chart shows the rates of contact by the research team, acceptance of an invitation to Vukuzazi, enrolment at the mobile health camp, and participation in the study components required to define four common and treatable diseases (HIV, tuberculosis, elevated blood pressure, and elevated blood glucose).

Eligible adolescent and adult residents, aged 15 years or older, from the Africa Health Research Institute demographic surveillance area in the uMkhanyakude district of KwaZulu-Natal, South Africa (figure 1, appendix 2 p 7) were invited to participate in this cross-sectional survey over an 18-month period (from May 25, 2018, to Nov 28, 2019). The district is typical of rural South Africa, with approximately 100% of individuals of Black African descent, 58% of adults unemployed, and 66% with access to piped water in their home (appendix 2 p 7).^14^ The University of KwaZulu-Natal Biomedical Research Ethics Committee, the London School of Hygiene & Tropical Medicine Ethics Committee, and the Partners Institutional Review Board approved the study protocol. All participants provided informed consent.

### Field and laboratory procedures

Individuals were visited at their homes (located using the geo-coordinates of their home) and invited to participate at a mobile health camp that moved through the study area during the study period. At the camp, research nurses administered questionnaires to assess the individual's history of HIV, tuberculosis, hypertension, and diabetes and to establish the presence of tuberculosis symptoms. Anthropometric and blood pressure measurements were done using the WHO STEPS protocol.^15^ Blood was obtained for measurement of glycosylated haemoglobin (glycated haemoglobin [HbA_1c_], measured using the VARIANT II TURBO Haemoglobin testing system [Bio-Rad, Marnes-la-Coquette, Paris, France]) and for HIV (Genscreen Ultra HIV Ag-Ab enzyme immunoassay [Bio-Rad]). Participants with a positive HIV immunoassay had a reflex HIV-1 RNA viral load test done (Abbott RealTime HIV-1 Viral Load [Abbott, IL, USA]). All non-pregnant participants were screened for tuberculosis with a digital chest x-ray. Sputum was obtained from participants reporting tuberculosis symptoms, all pregnant women, and all participants whose lung fields were abnormal on a chest x-ray, as established by real-time computer-assisted image analysis or by an experienced central radiologist,^16^, ^17^ and tested for *Mycobacterium tuberculosis* with an Xpert MTB/RIF Ultra test (Cepheid, Sunnyvale, CA, USA) and liquid mycobacterial culture (BACTEC MGIT 960 System [Becton Dickinson, Berkshire, UK]).

### Definition of diseases, disease control, and multimorbidity

Medical history and study measurements were used to define the presence of each of the four diseases and whether the disease was controlled (defined as diagnosed and optimally managed). Participants with a positive HIV immunoassay were defined as having HIV, and as having controlled disease if they were on antiretroviral therapy and had an HIV-1 RNA viral load of less than 40 copies per mL. Active tuberculosis was defined in those with sputum positive for *M tuberculosis* by Xpert MTB/RIF Ultra or liquid mycobacterial culture or those actively on tuberculosis treatment at the time of the survey, or both. Active tuberculosis was defined as controlled in participants who had started tuberculosis treatment before enrolment in Vukuzazi. Lifetime tuberculosis was defined as active tuberculosis, a history of tuberculosis treatment, or radiological evidence of previous tuberculosis as assessed by the radiologist, or a combination. Elevated blood pressure was defined as a mean systolic blood pressure of 140 mm Hg or more, diastolic blood pressure of 90 mm Hg or more at the Vukuzazi health camp, or having been previously diagnosed with hypertension and having taken antihypertensive medicines in the 2 weeks before enrolment. Of those who had taken anti-hypertensive medicines, those with a mean systolic blood pressure of less than 140 mm Hg and diastolic blood pressure of less than 90 mm Hg at the Vukuzazi health camp were defined as having controlled disease.^18^ Elevated blood glucose was defined as an HbA_1c_ of 6·5% or more, or having been previously diagnosed and having taken hypoglycaemic medicines in the 2 weeks before enrolment. Of those who had taken hypoglycaemic medicines, those with an HbA_1c_ of 6·5% or less were defined as having controlled disease.^19^ We constructed a multimorbidity scale that combined the control states of each of the four diseases (HIV, active tuberculosis, elevated blood pressure, and elevated blood glucose) to categorise participants into five states that ranged from healthiest to least healthy: (1) free of all measured diseases, (2) the presence of one controlled disease, (3) the presence of two or more controlled diseases, (4) the presence of one uncontrolled disease, and (5) the presence of two or more diseases, at least one of which was uncontrolled.

### Statistical analysis

Prevalence and 95% CIs for each of the diseases, their states of control, and multimorbidity categories were calculated overall and by sex and age groups. The age groups were 15–24, 25–44, 45–64, and ≥65 years. To account for the non-response of eligible members, estimates were weighted with the use of inverse probability weights, calculated as the inverse probability of participation by sex and age group. Continuous surface maps of disease and multimorbidity prevalence were generated with the use of a standard Gaussian kernel interpolation method (with a search radius of 3 km), which has been extensively used and validated in this population for mapping multiple HIV outcomes.^20^, ^21^ Analyses were done and some figures produced in R statistical software version 4·0·3 and the tidyverse package version 1·3·0. Additional figures were produced using Stata version 15·1·620 and maps were created using ArcGIS by Environmental Systems Research Institute version 10·5.

### Role of the funding source

The funders of the study had no role in study design, data collection, data analysis, data interpretation, or writing of the report.

## Results

A total of 17 118 individuals enrolled in Vukuzazi and 17 047 individuals completed all the study components required to define the four diseases and multimorbidity (figure 1). Women outnumbered men in the underlying population structure (appendix 2 p 9) and enrolled at higher rates across all age groups (table, appendix 2 p 10). Most participants resided in rural or peri-urban areas and the rate of unemployment among those in the labour force was high (table). Vukuzazi participants were older, more likely to be female, less highly educated, and had lower rates of employment than eligible non-participants (appendix 2 p 11).

**Table:** Population characteristics, disease prevalence, and multimorbidity

	**All (n=17 118)**	**Men (n=5500)**	**Women (n=11 618)**
**Age group (years)**			
15–24	4684 (27%)	2101 (38%)	2583 (22%)
25–44	5644 (33%)	1711 (31%)	3933 (34%)
45–64	4393 (26%)	1096 (20%)	3297 (28%)
≥65	2397 (14%)	592 (11%)	1805 (16%)
**Residence location**			
Urban	946 (6%)	292 (5%)	654 (6%)
Peri-urban	5509 (32%)	1820 (33%)	3689 (32%)
Rural	10 571/17 026 (62%)	3361/5473 (61%)	7210/11 553 (62%)
**Employment status**			
Unemployed*	3653/6516 (56%)	1169/2307 (51%)	2484/4209 (59%)
**Diseases**†			
HIV‡	34·2% (33·5–34·9)	24·8% (23·7–26·0)	40·0% (39·1–40·9)
Active tuberculosis§	1·4% (1·2–1·6)	1·8% (1·5–2·2)	1·1% (1·0–1·3)
Lifetime tuberculosis¶	21·8% (21·2–22·4)	23·4% (22·3–24·5)	20·8% (20·1–21·6)
Elevated blood glucose‖	8·5% (8·1–8·9)	4·9% (4·4–5·5)	10·7% (10·1–11·2)
Elevated blood pressure**	23·0% (22·4–23·6)	16·5% (15·5–17·4)	27·0% (26·2–27·8)
**Multimorbidity**††			
Healthy, no disease	47·9% (47·2–48·7)	61·3% (60·0–62·6)	38·5% (37·6–39·4)
One controlled disease	23·9% (23·3–24·6)	15·1% (14·2–16·1)	30·1% (29·3–31·0)
Two or more controlled diseases	2·3% (2·1–2·5)	1·1% (0·9–1·4)	3·1% (2·8–3·5)
One uncontrolled disease	16·4% (15·8–17·0)	16·3% (15·4–17·3)	16·4% (15·8–17·1)
Two or more diseases, at least one of which was uncontrolled	9·5% (9·1–9·9)	6·1% (5·5–6·7)	11·9% (11·3–12·5)

Data shown as n (%) or prevalence (95% CI).

* Unemployment calculated among members of the resident population in the labour force (n=6516).

† Prevalence weighted for non-response; weights calculated as the inverse probability of survey participation, in strata defined by age group and sex.

‡ HIV defined as a positive fourth generation antigen–antibody test.

§ Active tuberculosis defined as Vukuzazi sputum positive for *Mycobacterium tuberculosis* (by either GeneXpert Ultra or liquid culture, or both) or currently on treatment for clinically diagnosed tuberculosis, or both.

¶ Lifetime tuberculosis defined by a combination of active tuberculosis, self-reported current or past tuberculosis treatment, or radiological findings of previous tuberculosis disease on survey chest x-ray.

‖ Elevated blood glucose defined as a Vukuzazi glycated haemoglobin of 6·5% or more, or diagnosed with and on treatment for diabetes, or both.

** Elevated blood pressure defined as an average systolic blood pressure of 140 mm Hg or more or an average diastolic blood pressure of 90 mm Hg or more in the last two readings on Vukuzazi, or diagnosed with and on treatment for hypertension, or a combination.

†† Multimorbidity defined by the number and state of control for four diseases (HIV, active tuberculosis, elevated blood glucose, and elevated blood pressure).

**Figure 2:**
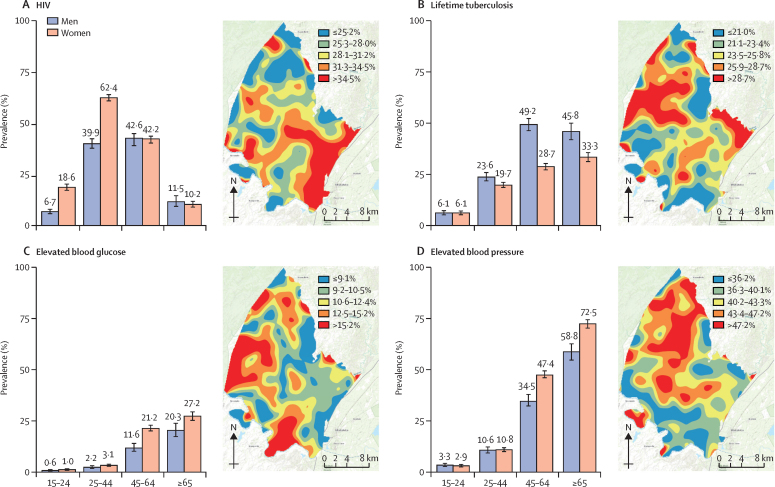
Population prevalence and spatial distribution of communicable and non-communicable diseases in rural KwaZulu-Natal, South Africa Population prevalence estimates by sex and age category (years) shown in the graphs, and continuous surface maps of the demographic surveillance area showing areas of lowest (blue) and highest (red) prevalence for HIV (A), lifetime tuberculosis (B), elevated blood glucose (C), and elevated blood pressure (D).

Among the four diseases assessed, HIV had the highest overall population prevalence (34·2%, 95% CI 33·5–34·9; table). Active tuberculosis was defined in 1·4% (1·2–1·6), lifetime tuberculosis in 21·8% (21·2–22·4), elevated blood pressure in 23·0% (22·4–23·6) and elevated blood glucose in 8·5% (8·1–8·9) of the population. HIV, elevated blood pressure, and elevated blood glucose were more common in women than in men, whereas active and lifetime tuberculosis were more common in men (table). HIV prevalence peaked in women aged 25–44 years, among whom more than half the population was infected (62·4%, 95 CI 60·8–63·9; figure 2A). In contrast, lifetime tuberculosis (figure 2B), elevated blood glucose (figure 2C), and elevated blood pressure (figure 2D) all increased with age in both men and women. Geospatial analysis showed the highest HIV prevalence in the southeastern region of the surveillance area, a peri-urban area that borders a national highway (figure 2A). Geospatial areas with highest prevalence of lifetime tuberculosis, elevated blood glucose, and elevated blood pressure showed little overlap with the area with highest prevalence of HIV (figure 2B–D).

**Figure 3:**
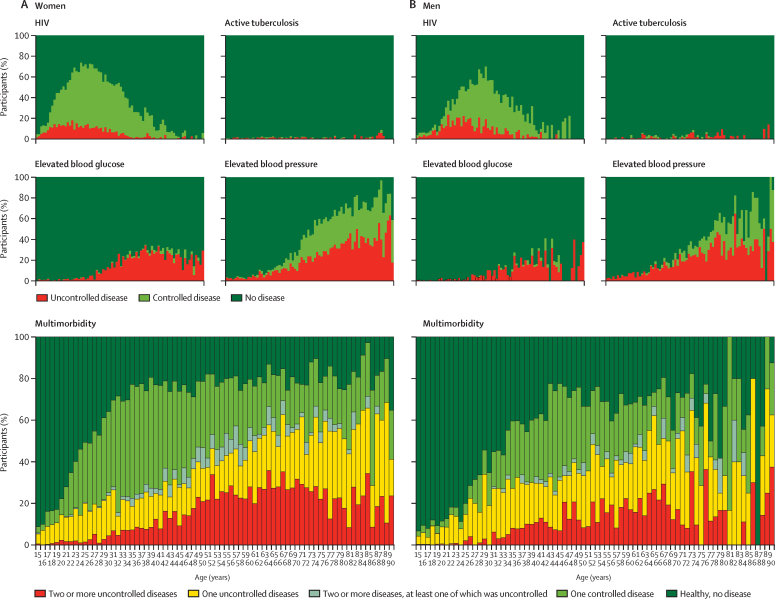
Disease control and multimorbidity Population prevalence of uncontrolled and controlled (A) HIV, (B) active tuberculosis, (C) elevated blood glucose, (D) elevated blood pressure, (E) multimorbidity by sex and age categories, and (F) a continuous surface map of the demographic surveillance area showing areas of lowest (blue) and highest (red) prevalence of the highest degree of multimorbidity (ie, the presence of two or more diseases, at least one of which was uncontrolled). M=male participants. F=female participants.

Among the four diseases, HIV had the highest rate of disease control, with the majority of participants who were HIV positive having an undetectable HIV-1 RNA viral load (78·1% [4526/5796], appendix 2 p 13). The 21·9% (1270/5796) of participants with uncontrolled disease included individuals newly diagnosed or not yet on antiretroviral therapy (12·9% [750/5796]) and those with detectable viraemia despite active antiretroviral therapy (9·0% [520/5796]). In contrast to HIV, the other three diseases had much lower rates of optimal disease control. Only 42·5% (1866/4395) of people with elevated blood pressure had well controlled disease, 29·6% (69/233) of people with active tuberculosis had been diagnosed and were receiving anti-tuberculosis treatment, and only 7·1% (117/1652) of those with elevated blood glucose had well controlled disease. Patterns of uncontrolled disease differed across sex and age groups, with the highest rates of uncontrolled HIV among men and women younger than 50 years (figure 3A), the highest rates of uncontrolled tuberculosis among men older than 50 years (figure 3B), and the highest rates of uncontrolled elevated blood glucose and blood pressure among men and women older than 50 years (figures 3C, D).

**Figure 4:**
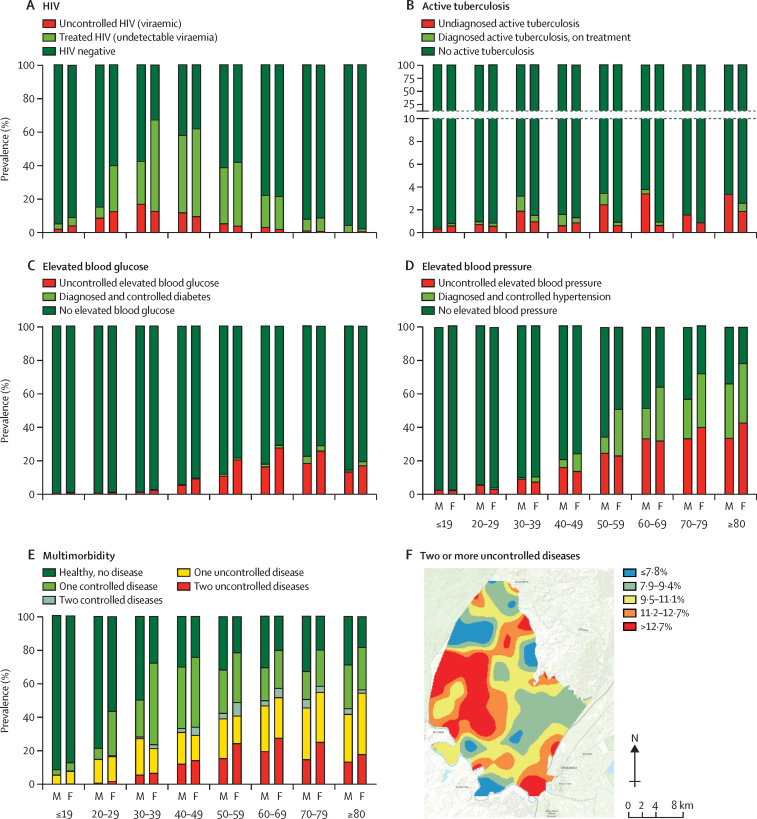
Multimorbidity across the lifespan Granular visualisation of the individual contributions of controlled or uncontrolled HIV, active tuberculosis, elevated blood glucose, and elevated blood pressure to multimorbidity across the adult lifespan of (A) women and (B) men who participated in Vukuzazi.

Multimorbidity analysis showed that 52·1% (95% CI 51·3–52·9) of the population had at least one active disease, 23·9% (23·3–24·6) had one disease that was optimally controlled, 2·3% (2·1–2·5) had two or more diseases all of which were optimally controlled, 16·4% (15·8–17·0) had one disease that was not optimally controlled, and 9·5% (9·1–9·9) had two or more diseases at least one of which was not optimally controlled (table, figure 3E, appendix 2 p 12). The healthiest state (zero diseases) was more frequent in men than women, whereas the least healthy state (the presence of two or more diseases, at least one of which was uncontrolled) was more frequent in women than men (table). Geospatial analysis identified two areas in the southeast and southwest of the demographic surveillance area with the highest prevalence of uncontrolled multimorbidity (figure 3F); notably, the southeastern area overlapped with the peri-urban area with highest HIV prevalence and the southwestern area corresponded to one of the most rural and least accessible parts of the demographic surveillance area. Granular visualisations of the four diseases illustrated the contribution of each disease and its state of control to multimorbidity over the adult lifespan (ages 15–90 years) of women (figure 4A) and men (figure 4B). Driven primarily by the HIV prevalence among women aged 30–40 years (67·5%, [95% CI 65·5–69·5]), by the age of 30 only 27·4% (25·5–29·4) of women were free from all four diseases. Despite their high prevalence, most of the diseases in women aged 30–50 years were controlled. In those older than 50 years, however, increasing rates of uncontrolled elevated blood glucose and elevated blood pressure contributed to high amounts of uncontrolled disease and multimorbidity (figure 4).

## Discussion

We found a high and overlapping prevalence of HIV, tuberculosis, elevated blood glucose, and elevated blood pressure in rural South Africa. Instead of the classic epidemiological transition in which societies shift from predominantly infectious to predominantly non-communicable burdens of disease over time, in this population we observed a convergence of infectious and non-communicable disease epidemics. Although HIV prevalence was high—with more than a third of the entire population and more than half of women aged 24–44 years positive for HIV— it was well controlled in approximately 80% of those affected. In contrast, most people with active tuberculosis, elevated blood pressure, and elevated blood glucose had disease that was not optimally controlled. Our results suggest that, despite the success of the HIV programme, the current health-care system fails to address the burden of multimorbidity in this population. Optimisation of population health in sub-Saharan Africa will require sustaining excellent HIV treatment programmes while strengthening multidisease approaches with targeted care for specific demographic groups.

Simultaneous measurement and analysis of these four common and treatable diseases highlighted the particularly heavy burden of disease borne by women (figure 4A). Our findings concur with other South African and sub-Saharan African studies that have found higher rates of HIV, hypertension, and diabetes in women.^22^, ^23^ Only in the youngest age strata (adolescents aged 15–19 years) were more than 80% of women free of disease. In this population, between the ages of 15 and 35 years, women had high HIV incidence, highlighting the persistent and urgent need to identify and implement interventions to prevent HIV infections.^24^ Because of the remarkably effective HIV care delivery system established in the region, more than 80% of women with HIV had an undetectable viral load on antiretroviral therapy.^25^ Yet, even as most women emerge from decades of high risk of HIV infection or with controlled HIV on lifelong antiretroviral therapy, by the age of 45 years, they transition to a period of high risk of comorbid diabetes and hypertension. Unlike HIV, these conditions are not currently addressed by a robust public health response, as shown by more than 64·1% of women older than 45 years having high blood glucose or high blood pressure; 68·4% of them poorly controlled. As such, our results support analyses from other South African settings in suggesting that the health of older women, in particular those who are not engaged in primary care for HIV infection, might be comparatively neglected.

Men had lower rates of HIV and non-communicable diseases but higher rates of both active and a history of tuberculosis. There are many reasons why these data should not be interpreted to mean that men have better health than women. First, our finding that nearly half of men older than 50 years had tuberculosis in their lifetime is concerning because there is emerging evidence that this is a risk factor for chronic lung disease,^26^ recurrent tuberculosis, and a decreased life expectancy.^27^ Additionally, men participated in Vukuzazi at lower rates than women and are known to be less often enrolled in HIV care programmes and to have lower life expectancy gains as a result.^28^ Finally, men are also at a greater risk for an early death in South Africa because of a higher burden of traumatic deaths, and thus our data could be susceptible to a survivor bias.^29^ In addition to highlighting the need to better diagnose and address tuberculosis and its sequelae in men, our results reinforce the need for novel delivery platforms to engage men in both health care and research to improve understanding of their health priorities.

The convergence of infectious and non-communicable disease epidemics poses notable challenges to the public health system. Despite calls to address the challenge of multimorbidity earlier in the course of the world's largest HIV epidemic, in South Africa and other countries in the same region, progress in establishing an excellent antiretroviral treatment programme in the public sector has outpaced that of diagnosing and treating chronic non-communicable diseases or fully integrating tuberculosis and HIV care.^30^, ^31^ Our findings suggest that the successes of the HIV care delivery system should be harnessed and applied to an integrated multidisease approach. Simultaneous epidemics that require lifelong treatments call for creative solutions that make use of non-traditional and community-based care systems to extend the reach of the health-care system.^32^ Approaches that include task-shifting to nurses and community health workers and integrating the management of multiple diseases have shown promise but require optimisation.^33^, ^34^ Decentralising care has the additional benefit of reducing clinic-based services and protecting individuals at a high risk of nosocomial exposures and complications in the face of emerging data from the COVID-19 pandemic, showing that poorly controlled multimorbidity predisposes individuals to additive morbidity and mortality risk. In addition, the health-care system needs to be able to provide access to the most vulnerable and those poorly engaged by conventional systems, including men and those residing in remote, rural areas.

The prevention of multimorbidity will require an improved understanding of the specific causal determinants of individual and overlapping diseases. Transdisciplinary research will be required to understand and address the contrasting demographic and geospatial distribution of infectious and non-communicable diseases, which might be influenced by biological, environmental, social, and health systems factors. Factors specific to sub-Saharan Africa, including the combined effects of childhood malnutrition, adult obesity, and high rates of maternal HIV infection, might have a unique effect on the long-term cardiometabolic health of the population.^8^ Although Africa has the highest genetic diversity of any continent and bears a disproportionate and unique burden of disease, it is under-represented among cohorts that assemble large multidimensional data and biorepositories to support modern genetic and molecular methods to advance public health.^35^, ^36^ The clinical, phenotypic, and geographical data presented here are nested within a longitudinal demographic surveillance cohort that has more than 15 years of individual and household structure data, which will allow for the spatially organised analysis of disease transmission and environmental exposures. The data presented here are augmented by a biorepository of over 250 000 biosamples that were collected and stored from more than 99% of the participants. The integrated analyses of genetic, biological, and social determinants of the four diseases measured here and the population health outcomes that will be assessed during ongoing demographic and health surveillance activities will enhance the understanding of current and emerging diseases, and allow for the emulation of scientific discovery similar to that of the UK BioBank and Framingham^36^, ^37^ in a rural South African population.

There are several limitations to our approach. Although we report a high rate of enrolment for a population-based multidisease study,^38^ non-systematic non-response, including lower rates of enrolment by men, might have resulted in bias during the data collection and skewed results. As is standard for population screening studies, we defined elevated blood pressure and blood glucose based on measurements done on a single day; additional longitudinal measurements are needed to establish diagnoses of hypertension and diabetes and clarify health system needs. Some of the definitions of disease included people with a self-reported history of diabetes, hypertension, or tuberculosis, which might incorporate diagnostic or reporting bias into our estimates. The prevalence of active tuberculosis might have been underestimated or overestimated because of the suboptimal diagnostic characteristics of sputum microbiological tests. Optimal therapeutic blood pressure and HBA_1c_ targets for anti-hypertensive and anti-hyperglycaemic treatments are topics of active investigation. For the purpose of this study, and to allow comparison between the four diseases, we defined optimal disease control using standard targets for blood pressure control and for people with newly diagnosed diabetes; however, the consideration of alternative targets for specific subpopulations (ie, people with other cardiac risk factors or older individuals) would be necessary to inform the design of interventional strategies. We assessed four common and treatable diseases in this study; however, important categories of disease, including cancer, chronic respiratory disease, mental health, and child health, were not measured in this study, limiting our ability to draw comprehensive conclusions about population health. In this initial effort to use mobile health camps to measure multiple diseases simultaneously, our selection of diseases was limited by practical and ethical considerations, and most importantly the need to consider the burden that community screening would place on the rural primary health-care system. Collaboration with the local and regional department of health personnel and across multiple medical disciplines was required to devise appropriate referral and treatment pathways for each disease measured. In the future, in partnership with local and provincial public health services, we aim to establish additional structures, thus allowing additional important categories of disease to be characterised in this population. Finally, the data presented are cross-sectional and so do not allow causal inference analysis of the effect of these diseases on a participant's eventual morbidity and mortality. Because this survey was situated within a demographic and health surveillance site that includes ongoing health information and verbal autopsy data, we expect to be able to contribute additional data on the longitudinal effects of these conditions and the effect of health systems interventions to ameliorate them in future reports.

In conclusion, we found converging epidemics of infectious and non-communicable diseases across the lifespan of adults in rural South Africa. Our findings of high rates of HIV requiring continuous access to daily medication, high rates of undiagnosed tuberculosis, low rates of optimally controlled non-communicable diseases, and complex patterns of multimorbidity highlight just some of the challenges in improving health for African populations with multiple intersecting epidemics. Our findings call for the development and evaluation of targeted public health programmes and scalable biomedical innovations to reduce the burden of specific diseases and multimorbidity to better population health. In the absence of improved disease prevention and control interventions, we expect the burden of multimorbidity to worsen as the generation of men and women most severely affected by the HIV epidemic ages over the next several decades. These challenges highlight the importance of investing in population science to define the interactions between infectious and non-communicable diseases and to harness the resulting knowledge to develop relevant biomedical and public health strategies to improve the health of African populations.

## Data sharing

Data and related documents, including the study protocol, informed consent forms, de-identified participant data, and a data dictionary defining each field, can be accessed via the Africa Health Research Institute Data Repository (please email RDMServiceDesk@ahri.org) after publication upon approval of the proposed analyses by the Vukuzazi Scientific Steering Committee and completion of a data access agreement.

## Declaration of interests

TN reports grants from the Wellcome Trust, the African Academy of Sciences, and the South African National Research Foundation, during the conduct of the study. ADG reports grants from the Wellcome Trust, during the conduct of the study. All other authors declare no competing interests.

## Supplementary Materials

isiZulu translation of the abstract
