# Supplementary material for: Convergence of infectious and non-communicable disease epidemics in rural South Africa: a cross-sectional, population-based multimorbidity study
Source: Lancet Glob Health. 2021 Jun 15;9(7):e967–76. doi: 10.1016/S2214-109X(21)00176-5 (PMC8220132; doi:10.1016/S2214-109X(21)00176-5)
Supplement: isiZulu translation of the abstract [file mmc1.pdf]

THE LANCET
Global Health

Supplementary appendix 1

This translation in isiZulu was submitted by the authors and we reproduce it as supplied. It has not been peer reviewed. *The Lancet*'s editorial processes have only been applied to the original in English, which should serve as reference for this manuscript.

Lokhu kuhumusha ngesiZulu kwethulwe ngababhali futhi siyikhiqiza kabusha njengoba kuhlinzekiwe. Akuzange kubuyekezwe ontanga. Izinqubo zokuhlela zeLancet zisetshenziswe kuphela koqobo lwesiNgisi, okufanele lusebenze njengesethenjwa salo mbhalo.

Supplement to: Wong EB, Olivier S, Gunda R, et al. Convergence of infectious and non-communicable disease epidemics in rural South Africa: a cross-sectional, population-based multimorbidity study. *Lancet Glob Health* 2021; **9:** e967–76.

Isifingqo (Abstract)

Isizinda (Background):

Sekubekhona inqubekelaphambili enkulu ekulashweni kweSandulela Ngculazi (HIV) e-Afrika engezansi kweSahara kodwa lusantengantenga ulwazi mayelana nezinga lezinye izimbangela zezifo ezibalulekile kanye nokuxhumana phakathi kwalezi zifo ne-HIV ezindaweni lapho khona i-HIV iningi khona emphakathini.

Izindlela (Methods):

Sisebenzise ingqalasizinda ebivele ikhona yokucubungula isimonjalo somphakathi (demography) kanye nezokubhekisiswa kwesimompilo somphakathi okuqhubekayo (health surveillance) ukuze siqagule izinga lobukhona be-HIV ebantwini kuleso sikhathi, ukusabalaliseka kwe-HIV endaweni (geospatial distribution), isibalo sabantu abaphethwe isifo sofuba (TB) nasebeke baphathwa isifo sofuba empilweni yabo, abanoshukela ophezulu, abanomfutho wegazi ophezulu kanye nabanenhlanganisela yazo zonke lezi zifo esifundeni esisendaweni yasemakhaya eNingizimu Afrika.

Okutholakele (Findings):

Kuhlolwe isamba sabantu abayizi-17 118 kuhlanganisa intsha nabantu abadala. Jikelele abantu abangamaphesenti angama-52.1 (95% CI 51.3-52.9) bebenesifo esisodwa esisabaphethe bese abangamaphesenti ayi-11.8 (95% CI 11.2-12.4) bebenezifo ezimbili kuya phezulu: 34.2% (95% CI 33.5-34.9) bebene-HIV, 1.4% (95% CI 1.2-1.6) bebenesifo sofuba esisaqhubeka, 21.8% (95% CI 21.2-22.4) abake baphathwa isifo sofuba ekuphileni kwabo, 8.5% (95% CI 8.1-8.9) bebenoshukela ophezulu kanti abangamaphesenti angama-23.0 (95% CI 22.4-23.6) bebenomfutho wegazi ophezulu. Uma sibheka ukulapheka nokulawuleka kwezifo, i-HIV ibilawuleke ngokwezinga eliphezulu (76.3%) bese lezi ezinye izifo bezilawuleke ngamazinga angaphansana: umfutho wegazi ophezulu (40.0%), isifo sofuba esiqhubekayo (31.3%) kanye noshukela ophezulu (6.9%). Ubukhona bezifo buhlukile uma kuqhathaniswa ngokobulili, ngokwamaqoqo eminyaka kanye nangokusabalaliseka kwabantu endaweni (geospatially): abesilisa bebenezinga eliphezulu lokuba nesifo sofuba esisaqhubeka nesike saba khona empilweni yonke, ekubeni abesifazane bebenamazinga aphezulu kakhulu e-HIV ebudaleni obuphakathi nendawo kanye namazinga aphakamayo enhlanganisela yezifo kanye nezifo ezingathathelani ezingalawulekile kahle emuva kweminyaka engama-50.

Ukuhunyuswa kokutholakele (Interpretation):

Sithole ukuhlangana kokubheduka kwezifo ezithathelanayo nezingathathelani emphakathini osendaweni yasemakhaya eNingizimu Afrika. Isandulela ngculazi besilapheke ngendlela egculisayo ebantwini kodwa isifo sofuba, ushukela ophezulu kanye nomfutho wegazi ophezulu bekungalaphekile kahle futhi bekungakaze kwatholakala ngendlela efanele kwabanye. Ezindaweni ezifana nalezi e-Afrika esezansi kweSahara kuyadingeka ukuthi kungenelele ezempilo zomphakathi (public health) ukuze kwenatshwe empumelelweni yokuhlolwa nokulashwa kwe-HIV kuhlanganiswe nohlelo lwezokwelapha oluzofaka phakathi ukuhlinzekwa kokunakekelwa kwenhlanganisela yezifo okubhekiswe ebantwini abathile emphakathini.

Uxhasomali (Funding):

Wellcome Trust, Bill and Melinda Gates Foundation, i-South African Department of Science and Innovation, South African Medical Research Council kanye ne-Population Research Infrastructure Network (SAPRIN)
