# Supplementary material for: Convergence of infectious and non-communicable disease epidemics in rural South Africa: a cross-sectional, population-based multimorbidity study
Source: Lancet Glob Health. 2021 Jun 15;9(7):e967–76. doi: 10.1016/S2214-109X(21)00176-5 (PMC8220132; doi:10.1016/S2214-109X(21)00176-5)
Supplement: Supplementary appendix 2 [file mmc2.pdf]

THE LANCET
Global Health

Supplementary appendix 2

This appendix formed part of the original submission and has been peer reviewed. We post it as supplied by the authors.

Supplement to: Wong EB, Olivier S, Gunda R, et al. Convergence of infectious and non-communicable disease epidemics in rural South Africa: a cross-sectional, population-based multimorbidity study. *Lancet Glob Health* 2021; **9:** e967–76.

Supplementary Appendix

This appendix has been provided by the authors to give readers additional information about their work.

Supplement to: Wong EB, Olivier S, Gunda R, Koole O, et al. Convergence of infectious and non-communicable disease epidemics in rural South Africa: a population-based multimorbidity study

Supplementary Appendix to Manuscript Entitled

Convergence of infectious and non-communicable disease epidemics in rural South Africa: a population-based multimorbidity study

Table of Contents

Vukuzazi Study Team.. 3

Supplemental Methods... 7

Eligibility and Recruitment 7
.
Informed consent... 7

Demographic data... 7

Health questionnaire 7

Blood pressure measurement.. 8

Sampling weights.. 8

Supplemental Figures and Tables

Figure S1. Population structure of eligible individuals and Vukuzazi participants......... 9

Figure S2. Outcome of recruitment efforts by age and sex... 10

Table S1. Demographic characteristics of the eligible population, participants and non-participants.. 11

Table S2. Combinations of diseases contributing to multimorbidity in Vukuzazi……...12

Table S3. Details of control states for four diseases assessed in Vukuzazi……………..13

References 14

Vukuzazi Team: Staff who significantly contributed to the implementation and conduct of Vukuzazi.

***** Denotes team members who were closely involved with the design, implementation and oversight of Vukuzazi.

Name	Role
*Deenan Pillay	Principal Investigator (2017-2019)
*Willem Hanekom	Principal Investigator (2019-present)
*Emily Wong	Co-Principal Investigator
*Mark Siedner	Co-Principal Investigator
*Olivier Koole	Co-Principal Investigator (2017-2019)
*Thumbi Ndung'u	Co-investigator
*Thandeka Khoza	Co-investigator (2019-present)
*Kobus Herbst	Co-investigator
*Kathy Baisley	Co-investigator
*Janet Seeley	Co-investigator
*Alison Grant	Co-investigator
*Resign Gunda	Programme Manager
*Ashmika Surujdeen	Study Coordinator
*Theresa Smit	Head: Diagnostic Research
*Dickman Gareta	Head: Research Data Management
*Day Munatsi	Head: Research Data Systems
*Ngcebo Mhlongo	Study Physician
*Sanah Bucibo	Lead Nurse
*Tshwaraganang Modise	Research Data Manager
*Stephen Olivier	Statistician
*Gregory Ording-Jespersen	Laboratory Data Supervisor
*Innocentia Mpofana	Diagnostic Laboratory Manager
*Jaco Dreyer	Senior Research Data Manager
*Siyabonga Nxumalo	Research Data Manager
*Khadija Khan	Biorepository Manager
*Zizile Sikhosana	Somkhele Laboratory Supervisor
*Sashen Moodley	Microbiology Laboratory Supervisor
*Hollis Shen	Head: Exploratory Research Division
Kennedy Nyamande	Pulmonology Consultant
Mosa Suleman	Pulmonology Consultant
Jaikrishna Kalideen	Radiologist
Ramesh Jackpersad	Radiologist
Kgaugelo Moropane	Radiographer
Boitsholo Mfolo	Radiographer
Khabonina Malomane	Radiographer
Hlolisile Khumalo	Nursing Manager
Nompilo Buthelezi	Training Coordinator
Nozipho Mbonambi	Professional Nurse
Hloniphile Ngubane	Professional Nurse

Thokozani Simelane	Professional Nurse
Khanyisani Buthelezi	Professional Nurse
Sphiwe Ntuli	Professional Nurse
Nombuyiselo Zondi	Professional Nurse
Siboniso Nene	Professional Nurse
Bongumenzi Ndlovu	Enrolled Nurse
Talente Ntimbane	Enrolled Nurse
Mbali Mbuyisa	Enrolled Nurse
Xolani Mkhize	Enrolled Nurse
Melusi Sibiya	Enrolled Nurse
Ntombiyenkosi Ntombela	Enrolled Nurse
Mandisi Dlamini	Enrolled Nurse
Hlobisile Chonco	Enrolled Nurse
Hlengiwe Dlamini	Enrolled Nurse
Doctar Mlambo	Enrolled Nurse
Nonhlanhla Mzimela	Enrolled Nurse
Zinhle Buthelezi	Enrolled Nurse
Zinhle Mthembu	Enrolled Nurse
Thokozani Bhengu	Enrolled Nurse
Sandile Mthembu	Enrolled Nurse
Phumelele Mthethwa	Enrolled Nurse
Zamashandu Mbatha	Enrolled Nurse
Welcome Petros Mthembu	Enrolled Nurse
Anele Mkhwanazi	Clinical Research Assistant Supervisor
Mandlakayise Zikhali	Clinical Research Assistant Supervisor
Phakamani Mkhwanazi	Clinical Research Assistant
Ntombiyenhlanhla Mkhwanazi	Clinical Research Assistant
Rose Myeni	Clinical Research Assistant
Fezeka Mfeka	Clinical Research Assistant
Hlobisile Gumede	Clinical Research Assistant
Nonceba Mfeka	Clinical Research Assistant
Ayanda Zungu	Clinical Research Assistant
Hlobisile Gumede	Clinical Research Assistant
Nonhlanhla Mfekayi	Clinical Research Assistant
Smangaliso Zulu	Clinical Research Assistant
Mzamo Buthelezi	Clinical Research Assistant
Senzeni Mkhwanazi	Clinical Research Assistant
Mlungisi Dube	Clinical Research Assistant
Philippa Mathews	Clinical Governance
Siphephelo Dlamini	AHRI Nursing Manager
Hosea Kambonde	IT Systems Developer
Lindani Mthembu	Information Technology Assistant
Seneme Mchunu	Information Technology Assistant
Sibahle Gumbi	Research Admin Assistant
Tumi Madolo	Research Data Manager

Thengokwakhe Nkosi	Driver
Sibusiso Mkhwanazi	Driver
Sibusiso Nsibande	Driver
Mpumelelo Steto	Driver
Sibusiso Mhlongo	Driver
Velile Vellem	Driver
Pfarelo Tshivase	Driver
Jabu Kwinda	Driver
Bongani Magwaza	General Worker
Siyabonga Nsibande	General Worker
Skhumbuzo Mthombeni	General Worker
Sphiwe Clement Mthembu	General Worker
Antony Rapulana	Laboratory Technologist
Jade Cousins	Laboratory Technologist
Thabile Zondi	Laboratory Technologist
Nagavelli Padayachi	Laboratory Technologist
Freddy Mabetlela	Laboratory Technologist
Simphiwe Ntshangase	Laboratory Technician/LIMS Administrator
Nomfundo Luthuli	Laboratory Technician
Sithembile Ngcobo	Laboratory Technologist
Kayleen Brien	Laboratory Technologist
Sizwe Ndlela	Laboratory Technician
Nomfundo Ngema	Laboratory Technician
Nokukhanya Ntshakala	Laboratory Technician
Anupa Singh	Laboratory Technician
Rochelle Singh	Laboratory Technician
Logan Pillay	Laboratory Technician
Kandaseelan Chetty	Laboratory Technician
Ashentha Govender	Laboratory Technician
Pamela Ramkalawon	Laboratory Research Technician
Nondumiso Mabaso	Laboratory Intern
Kimeshree Perumal	Laboratory Intern
Senamile Makhari	Biorepository Laboratory Technician
Nondumiso Khuluse	Biorepository Laboratory Technician
Nondumiso Zitha	Biorepository Research Assistant
Hlengiwe Khathi	Biorepository Research Assistant
Mbuti Mofokeng	Clinical Specimen Driver/Laboratory Assistant
Nomathamsanqa Majozi	Public Engagement
Nceba Gqaleni	Public Engagement
Hannah Keal	Communications
Phumla Ngcobo	Communications
Costa Criticos	Operational Oversight
Raynold Zondo	Operational Oversight
Dilip Kalyan	Operational Oversight
Clive Mavimbela	Operational Oversight

Anand Ramnanan	Procurement
Sashin Harilall	Grants Office

Supplemental Methods

Additional Methodological Details

Eligibility and Recruitment. Eligibility criteria included age of at least 15 years on the day of recruitment and ongoing residency in the southern part of the AHRI demographic surveillance area. Eligible individuals were enumerated from the surveillance database on a weekly basis to ensure that most up-to-date data were used to reduce non-contact resulting from migration and death. A resident member of a household was defined as an individual who had slept the majority of nights in the preceding 4 months in the homestead occupied by the household. Study field workers visited households to explain the Vukuzazi survey, provide a written description of the study, and invite eligible household members to participate. Survey field workers provided registered invitation cards to potential participants in person or by proxy (issued to a household informant for other eligible household residents who were not home at the time of the recruiter's visit). Individuals in possession of registered invitation cards were eligible to attend the health camp on any subsequent date.

Informed consent. Upon presentation to the health camp, potential participants received additional written and verbal explanation of survey procedures, underwent assessment of capacity, individual informed consent and enrollment into the survey. All participants completed a formal written informed consent process that included consent for disease testing and return of results, storage and future use of biobanked specimens, permission for genetic testing in the future, and permission to be re-contacted for additional sub-studies and longitudinal follow-up. Adolescent participants who were younger than 18 years of age additionally required written assent for participation from their parent or guardian.

Demographic data. Demographic information about Vukuzazi participants and non-participants (including marital status, employment status, level of education attained, location of residence and household access to running water) were obtained from the most recent demographic survey. Membership in the labour force and employment status were defined using the strict definition of unemployment as defined by Statistics South Africa.(1)

Health questionnaire: At the Vukuzazi health camp, a study nurse administered a personal health history questionnaire that included personal history of HIV (including date and result of most recent HIV test and personal history of antiretroviral therapy), personal history of tuberculosis (including date of most recent TB treatment, current TB treatment status and ascertainment of the presence and duration of the four cardinal symptoms of TB (cough, fever, night sweats and weight loss) as designated in WHO TB Prevalence Survey methodology,(2) personal history of diabetes (whether the condition had been

diagnosed and whether the participant was actively receiving antidiabetes therapy), and personal history of hypertension (whether the condition had been diagnosed and whether the participant was actively receiving antihyptertensive therapy).

Blood pressure measurement. Systolic and diastolic blood pressure were measured using automated devices (Welch Allyn) three times at 15 minute intervals when participants were comfortably seated and had been sitting with their legs uncrossed and feet on the ground for at least 15 minutes prior to the first measurement, according to the WHO STEPS protocol.(3) Small adult, adult, large adult and extra-large adult blood pressure cuffs were selected based on measured arm circumference (22-26cm, 27-34cm, 35-44cm, 45-52cm respectively). The average of the second and third systolic and diastolic measurements was used to define blood pressure for the analysis.

Statistical approaches. Inverse probability weights, to account for study non-participation, were calculated as the inverse probability of participation in the study, using a logistic regression model including all eligible individuals in the catchment area, with covariates for age groups (in 10 year bands) and sex. The inverse of the predicted probability from the regression model was used as the weight. Unweighted frequencies of multimorbidity categories in 1 year age bands were used to visualize the spectrum of health and across the adult lifespan. Since all resident individuals in the surveyed area of the PIPSA were eligible for the study, sampling weights were not applied in the calculation of population prevalence estimates. Continuous surface maps of disease and multimorbidity prevalence were generated using kernel interpolation method with a moving two-dimensional Gaussian kernel of a 3-km search radius which has been extensively validated in our setting.(4-7) The search radius was determined using a spatial variogram (constructed using HIV prevalence estimates aggregated by administrative ward), which showed there to be clear spatial dependence in the resulting HIV prevalence estimates within a distance of 3km whereafter the dependence ceased. The large sample size for this study meant that the resulting estimates were robust to the effects of random noise. For consistency and ease of comparison we applied the same technique and search radius to all four diseases.

Supplementary Figures and Tables

Figure S1. Population structure of eligible individuals and Vukuzazi participants.
Population pyramid of eligible residents of the AHRI demographic surveillance area (no outline) and of Vukuzazi participants (black outline) by age and sex strata.

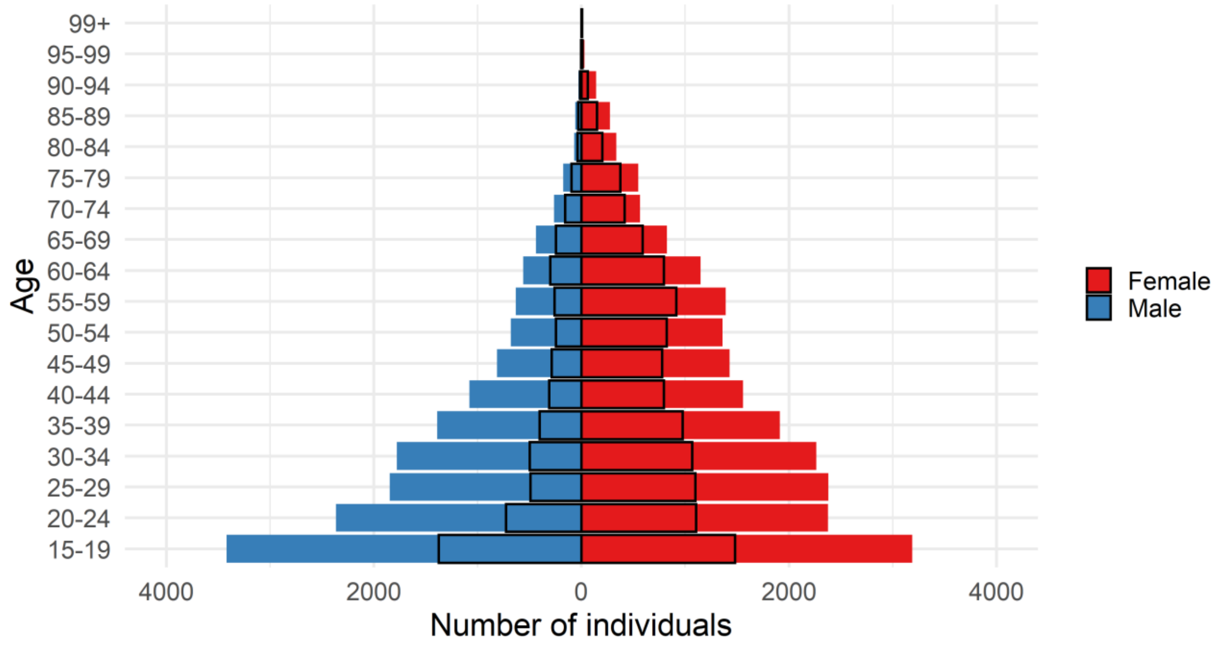

Figure S2. Outcome of recruitment efforts by age and sex. The percentage of eligible population whom the study team were unable to contact (blue), who refused further participation after hearing about the nature of the study (purple), who accepted an invitation card but did not attend Vukuzazi (light green) and who attended the Vukuzazi mobile health camp (dark green).

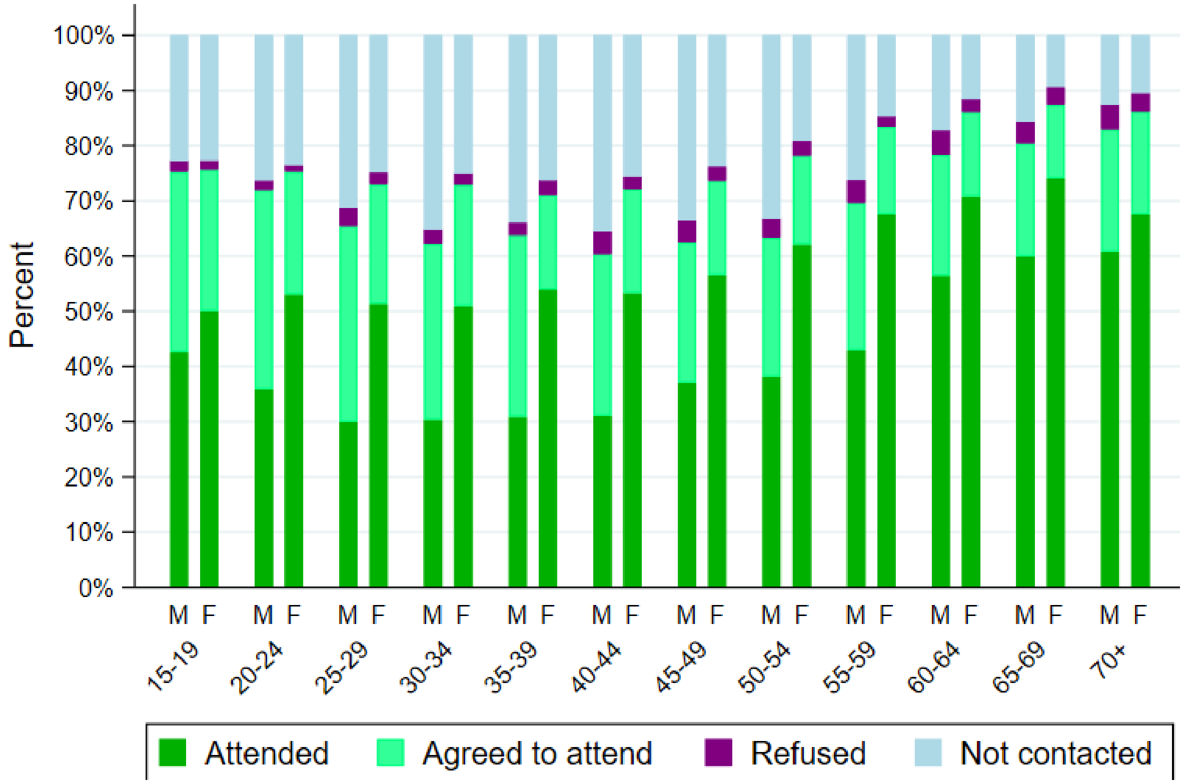

Table S1. Demographic characteristics of the eligible population, participants and non-participants.

	Eligible population (N=34721)	Vukuzazi participants (N=17118)	Vukuzazi non-participants (N=17603)	p-value[1]
**Age group**	**n=34721**	**n=17118**	**n=17603**	<0.000
<25	10294 (29.6%)	4684 (27.4%)	5610 (31.9%)	
25-34	7523 (21.7%)	3155 (18.4%)	4368 (24.8%)	
35-44	5612 (16.2%)	2489 (14.5%)	3123 (17.7%)	
45-54	4102 (11.8%)	2123 (12.4%)	1979 (11.2%)	
55-64	3617 (10.4%)	2270 (13.3%)	1347 (7.7 %)	
>65	3573 (10.3%)	2397 (14.0%)	1176 (6.7 %)	
**Gender**	n=34721	n=17118	n=17603	<0.000
Male	14402 (41.5%)	5500 (32.1%)	8902 (50.6%)	
Female	20319 (58.5%)	11618 (67.9%)	8701 (49.4%)	
**Education**	n=34580	n=17076	n=17504	<0.000
None	8076 (23.4%)	4320 (25.3%)	3756 (21.5%)	
Less than complete secondary	11402 (33.0%)	6409 (37.5%)	4993 (28.5%)	
Complete seconary and above	15102 (43.7%)	6347 (37.2%)	8755 (50.0%)	
**Marital Status**	n=29803	n=14839	n=14964	<0.000
Single	7566 (25.4%)	3567 (24.0%)	3999 (26.7%)	
Married/Living as married	19041 (63.9%)	9185 (61.9%)	9856 (65.9%)	
Seperated/Widowed/Divorced	3196 (10.7%)	2087 (14.1%)	1109 (7.4 %)	
**Employment Status[2]**	n=15746	n=6516	n=9230	<0.000
Unemployed[3]	6959 (44.2%)	3653 (56.1%)	3306 (35.8%)	
Employed	8787 (55.8%)	2863 (43.9%)	5924 (64.2%)	
**Residence Location**	n=34531	n=17026	n=17505	<0.000
Urban	2862 (8.3 %)	946 (5.6 %)	1916 (10.9%)	
Peri-urban	11720 (33.9%)	5509 (32.4%)	6211 (35.5%)	
Rural	19949 (57.8%)	10571 (62.1%)	9378 (53.6%)	

[1]Chi-squared test of difference in each demographic category between Vukuzazi participants and non-participants. [2]Employment status calculated among members of the resident population in the labour force. [3]Department of Statistics South Africa's strict definition of unemployment (Ref 1).

Table S2. Combinations of diseases contributing to multimorbidity in Vukuzazi. Table includes all participants who enrolled between May 2018-December 2019 (n = 17,118). Glu = Elevated blood glucose; BP = elevated blood pressure; Missing = participants who did not complete disease screening activities for all four diseases.

Disease combinations	N	percent
No disease	7553	44.12
Glu	385	2.25
Glu_BP	881	5.15
Glu_BP_TB	14	0.08
Glu_TB	9	0.05
HIV	4380	25.59
HIV_Glu	162	0.95
HIV_Glu_BP	194	1.13
HIV_Glu_BP_TB	1	0.01
HIV_Glu_TB	6	0.04
HIV_BP	945	5.52
HIV_BP_TB	15	0.09
HIV_TB	93	0.54
BP	2319	13.55
BP_TB	26	0.15
TB	69	0.4
Missing	66	0.39

Table S3. Details of control states for four diseases assessed in Vukuzazi.

	HIV		Elevated blood glucose		Elevated blood pressure		Active tuberculosis	
	N	percent	N	Percent	N	Percent	N	Percent
Controlled (Diagnosed, On optimal treatment)	4526	78.1%	117	7.1%	1866	42.5%	69	29.6%
Uncontrolled (Newly diagnosed)	599	10.3%	415	25.1%	764	17.4%	164	70.4%
Uncontrolled (Previously diagnosed, Not on treatment)	151	2.6%	559	33.8%	704	16.0%	0	0
Uncontrolled (Previously Diagnosed, On suboptimal treatment)	520	9.0%	561	34.0%	1061	24.1%	0	0
Total	5796	100,0%	1652	100.0%	4395	100.0%	233	100.0%

References

1. Quarterly Labour Force Survey, Statistical Release Pretoria, South Africa: Department of Statistics South Africa 23 June 2020.
2. Tuberculosis prevalence surveys: a handbook. Geneva, Switzerland: World Health Organization March 2011.
3. The WHO STEPwise approach to noncommunicable disease risk factor surveillance (STEPS) - Instrument. Geneva, Switzerland: World Health Organization. Contract No.: v3.2.
4. Tanser F, Barnighausen T, Cooke GS, Newell ML. Localized spatial clustering of HIV infections in a widely disseminated rural South African epidemic. International journal of epidemiology. 2009;38(4):1008-16.
5. Tanser F, Vandormael A, Cuadros D, Phillips AN, de Oliveira T, Tomita A, et al. Effect of population viral load on prospective HIV incidence in a hyperendemic rural African community. Science translational medicine. 2017;9(420).
6. Vandormael A, Cuadros D, Kim HY, Barnighausen T, Tanser F. The state of the HIV epidemic in rural KwaZulu-Natal, South Africa: a novel application of disease metrics to assess trajectories and highlight areas for intervention. International journal of epidemiology. 2020;49(2):666-75.
7. Waller LA, Gotway CA. Applied Spatial Statistics for Public Health Data: John Wiley & Sons; 2004.
